# Ethnological approach to acorn utilization in prehistory: A case study of acorn mook making in South Korea

**DOI:** 10.3389/fpls.2022.996649

**Published:** 2022-10-18

**Authors:** Ting An, Mengxia Tang, Juhui An

**Affiliations:** School of Art and Archaeology, Zhejiang University, Hangzhou, China

**Keywords:** acorn, *chaîne opératoire*, ethnological approach, prehistory, mook making

## Abstract

Acorn remains are reported from prehistoric sites across the world. Acorn is argued to have been an important food resource for human beings in prehistory. However, relevant research is still limited and it is often difficult to recognize archaeological remains relating to acorn utilization. The *Chaîne Opératoire* of acorn utilization is yet to be addressed. Such is of great significance to the study of human subsistence strategy in pre-agricultural period and moreover the origin of agriculture. By conducting a case study of ‘mook making’ using acorns in Yongdong-gun, Chungcheongbuk-do, South Korea, the current paper explores the *Chaîne Opératoire* of acorn utilization in prehistory using an ethnological approach. We draw attention to the laborious nature of acorn processing and to different methods of acorn processing due to different species and culinary tradition. Our case study also brings new insights into archaeological interpretations of acorn remains from prehistoric sites.

## Introduction

The term acorn refers to the fruit of genera *Quercus*, *Cyclobalanopsis*, *Castanopsis* and *Lithocarpus* of the family *Fagaceae*. Acorns are often described as being rich in starch and used as food or fodder (Hu et al., 2000). They are generally round or oblong in shape, and contain a kernel wrapped in a tough and smooth leathery shell with a cup-shaped cupule. The identification of acorn species is sometimes discussed by archaeobotanists (e.g. Yang, 1979; Zhao and Zhang, 2009; Fuller et al., 2011) while in more cases, archaeological excavation reports tend to use the term of acorn without differentiation (e.g. Wang et al., 2001; Sun et al., 2007; Li et al., 2020). Acorns have a long history of human utilization in many parts of the world, with archaeological evidence traced back to more than 700,000 years ago (Mason, 1992; Melamed et al., 2016). Particularly in East Asia, the widespread findings of acorn remains, especially large quantities from sites such as Kuahuqiao and Tianluoshan in Southeast China, Bibong-ri in South Korea, Sobata and Saka-no-shita in Japan, suggest that acorns might once have played a very important part in the diet of human beings (Saga Prefecture Education Committee, 1977; Takahashi and Hosoya, 2002; ZPICRA, 2004; Sun et al., 2007; Lee, 2008; Fuller et al., 2011). The significance of acorn utilization as a subsistence strategy in pre-agricultural period is often discussed, which is considered to be related to the origin of agriculture (McCorriston, 1994; Barlow and Heck, 2002; Fuller and Qin, 2010). Recognizing the remains and residues concerning acorn processing from archaeological sites accordingly becomes crucial. This paper assesses the *Chaîne Opératoire* (or operational chain) of acorn utilization in modern East Asia so as to provide new insight for research on acorn findings.

Previous studies are largely focused on each individual procedure of acorn use, such as acorn collection (Qin et al., 2010), acorn storage (Im and Lee, 2010) and culinary tradition (Liu et al., 2010a; Zhang, 2011; Stevens and McElreath, 2015; Yao et al., 2016). There have been a number of studies addressing the *Chaîne Opératoire* of acorn processing (e.g. Rosenberg, 2008; Hosoya, 2011; Ahn, 2012; Primavera and Fiorentino, 2013; Chen and Chen, 2019; Wang and Jiang, 2022). Based on existing literature and first or second-hand ethnographic sources, these studies explicit the operational chain of acorn processing in various context. Some scholars further relate the ethnographic information to interpretation of acorn remains in archaeological sites (Rosenberg, 2008; Hosoya, 2011; Primavera and Fiorentino, 2013; Chen and Chen, 2019; Wang and Jiang, 2022). Nevertheless, existing studies call for more detailed observation of the acorn use operational chain.

In light of this, this paper attempts to explore the *Chaîne Opératoire* of acorn utilization using an ethnological approach. Acorn mook, a type of jelly made from acorn, is consumed in both South Korea and China (*cazhi doufu* or *xiangzi doufu* in Chinese). The recipe of mook, not necessarily made of acorn but mung bean, buckwheat, etc., already appeared in the 18^th^ century literature (Cha et al., 2008). The processing method of acorn mook in particular is recorded in Korean historical document *Chosun Yori-Jebup* which was dated a hundred years old (Bang, 1917), and is similar to what is still practiced today. Therefore, we take the culinary tradition of ‘acorn mook making’ in Yongdong-gun, Chungcheongbuk-do, South Korea as a case study, to explicate the operational chain of acorn use and to discuss about associated potential archaeological and archaeobotanical evidences in prehistoric sites.

## Case study of acorn mook production in modern South Korea

**Figure 1:**
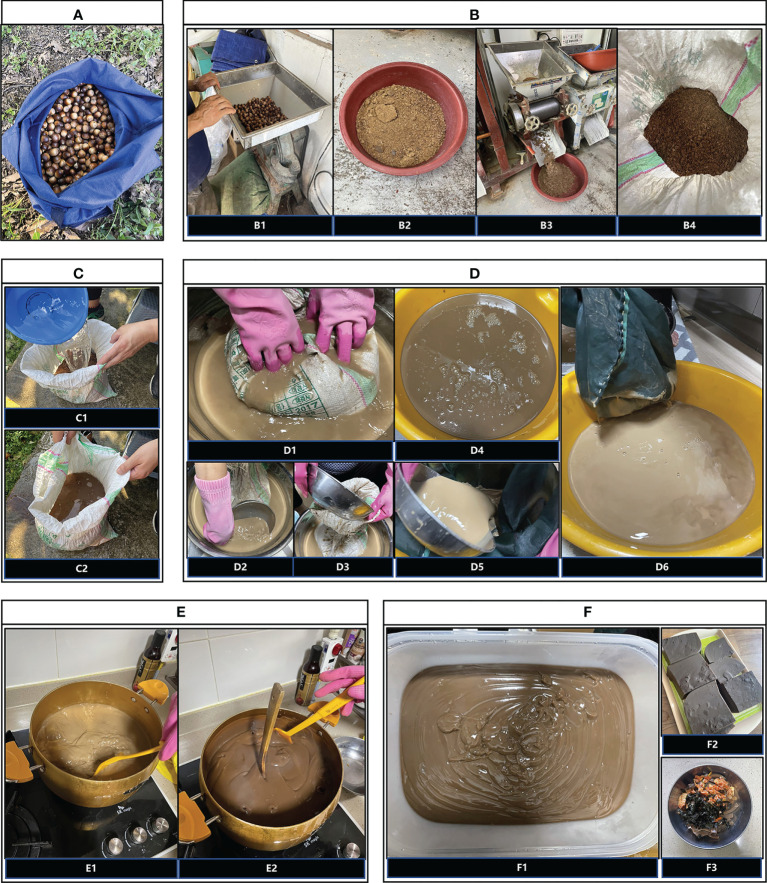
Ethnological study of ‘mook-making’ process in Chungcheongbuk-do, South Korea. **(A)** Collected acorns. **(B)** Grinding acorns using the modern crushing machines. B1. First grinding. B2. Acorn powder after the first grinding. B3. Second grinding. B4. Acorn powder after the second grinding. **(C)** Rinsing and filtering the ground acorn powder repetitively. **(D)** Starch-shell separation to extract acorn starch. D1-D3. Kneading constantly. D4. Liquid after the first filtering. D5. Pouring liquid into another bag for a second filtering. D6. Liquid after the second filter. **(E)** Boiling the acorn starch. E1. Acorn starch before boiling. E2. Acorn starch after boiling. **(F)** Solidification and serving. F1. 1,600ml acorn starch in a plastic mould. F2. Six mook blocks. F3. Mook-sabal.

In October 2021, we visited a local lady (hereafter Mrs W) based in Chungcheongbuk-do, South Korea and recorded the whole process of ‘acorn mook making’ (**Figure 1**).

### Collecting

**Figure 2:**
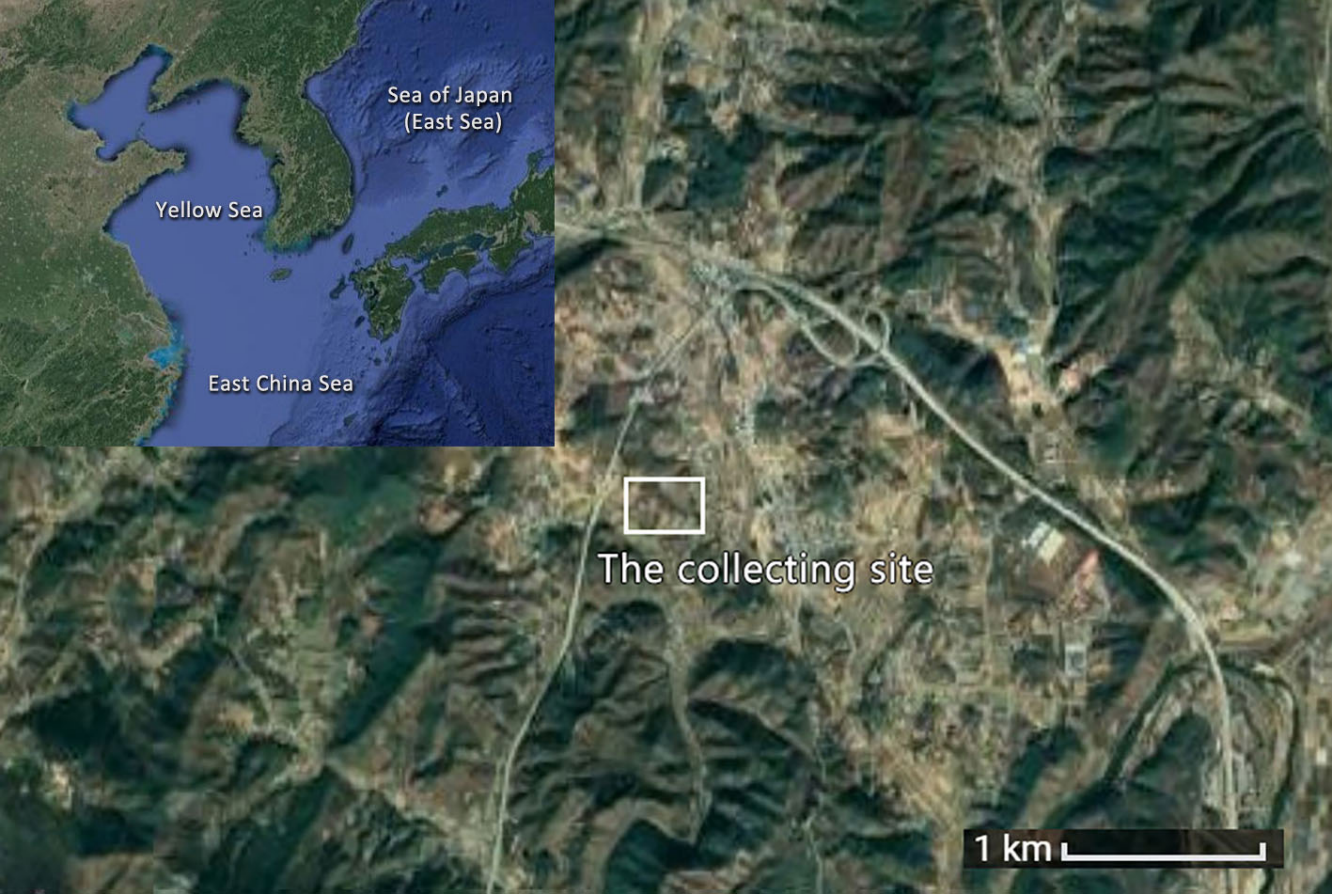
Geographical location of acorn collection site.

The acorn collection site in the case study is located on a hilly area in Chungcheongbuk-do, Republic of Korea, at an altitude of approximately 167m (**Figure 2**). The area is dominated by oak trees and is within five minutes’ walking time of nearby settlements.

Mrs W, her friend and her granddaughter spent a week collecting acorns, about an hour at noon each day and stored the collected acorns in a plastic basket at home. The acorns collected were from sawtooth oaks (*Quercus acutissima*), which are about 20-25 meters high. She and her companions chose the mature acorns which had fallen off the trees. After a week’s accumulation, the total number of acorns collected accounted for one third of the volume of a 30cm x 30cm bag and was around 2 kilograms.

### Grinding

The process of grinding took place at the grinding factory in the market of Yongsan-myeon, Yeongdong-gun. Mrs W and her granddaughter carried the bag with acorns from the collection site to the factory, which was about ten minutes’ drive. Acorns collected were ground twice using the machine operated by the factory owner. The owner poured the whole bag of acorns into the upper part of the machine for the first grinding. Acorn powder with a few lumps was then discharged from the lower outlet after ten seconds. Before the second grinding, Mrs W quickly crushed the large lumps with her own hands. The second grinding took place in another machine and was operated in a similar way. It took a little longer, about a minute. Finally, she used a plastic ladle to scoop the ground acorn flour into a sack provided by the grinding factory, so as to facilitate the outflow of water during the next process, namely rinsing and filtering.

According to Mrs W, when there were no modern crushing machines, there were two tools for manual grinding, stone mill and foot mortar. Comparing manual grinding methods with machine grinding, the latter was considered the most efficient, followed by foot mortar method, while stone mill method has the lowest efficiency. More specifically, the amount of acorn ground by the machine in ten minutes would take two to three hours using foot mortar and at least half a day using a stone mill. In addition, acorn powder ground by machine tends to be finer, producing a larger quantity of mook. By contrast, with manual grinding, especially stone mill grinding, the powder obtained is often less fine with large lumps, leading to less efficient utilization of acorns. Three types of grinding methods are compared in **Table 1**.

**Table 1:** Comparison of three types of grinding methods.

Method	Machine grinding	Stone mill method	Foot mortar method
**Efficiency**	high	low	moderate
**Fineness of the powder**	small	large	moderate
**Amount of mook produced from the same amount of acorns**	large	small	moderate

### Rinsing and filtering

Mrs W mentioned that, in some families, acorns might be soaked in water for a week before the next process. She also emphasized that rinsing and filtering is crucial for the taste of the final product. The longer acorn powder is rinsed and filtered, the better it would taste. In order to remove tannins and reduce the bitterness of the mook, her granddaughter spent 3.5 hours rinsing the ground acorn powder in the sack using running water. Depending on the amount of acorn powder, the rinsing time varies, according to the young lady. She rinsed with a connected hose on the gravel path at the edge of Mrs W’s farm. Each time she made sure that the water completely submerged the acorn powder, and then waited for the water to run out. She did the same task repetitively. The whole job was completed when the water coming out of the sack was no longer red and appeared clear.

### Starch-shell separation

After rinsing and filtering, Mrs W and her granddaughter took the entire sack of acorn powder home for the rest of the procedure. Because the acorn flour was dripping, they put the sack in a plastic basket while driving home. The flour, along with the sack, was rubbed by hand in a basin. According to Mrs W, the purpose of starch-shell separation is to extract acorn starch, and the constant kneading is to separate the starch from the powder. The young lady poured water into the sack containing the acorn powder and spent around fifteen minutes kneading with her hands while the liquid flowed into a basin. The residue left in the sack was then thrown away.

A second filtering process was then carried out with another weaving bag in a similar way. The bag used for the second time was common in Korean households, often used for making broths, with smaller aperture size. It took a similar amount of time, resulting in a liquid that filled approximately one small basin. After the second filter, the residue was removed again and the brown liquid with white acorn starch particles (visible to naked eyes) in the basin remained. Mrs W added that, “It doesn’t matter how much or how little liquid there is, whether it’s thick or thin. It’s white acorn starch that really matters”.

### Boiling

The purpose of boiling is to evaporate the water. The young lady boiled the acorn starch in a pot and kept stirring to avoid the lower part of the paste from being burnt. The paste slowly became gluey, smaller in volume and darker in color. The whole process took about twenty minutes when an inserted spoon could stand upright without falling over. There was about 1,600 ml of acorn starch in the end.

### Solidification and serving

When the boiling was completed, the acorn starch was poured into a plastic mould and was left to solidify at room temperature. It took three to four hours for the acorn paste to solidify. Then it was cut into six blocks by approximately 8cm × 8cm × 4cm.

The young lady introduced two mook recipes in South Korea: dipped with seasoned soy sauce or made with kimchi into a kind of soup which was called mook-sabal/묵사발.

## Discussion

### *Chaîne Opératoire* of acorn use

**Figure 3:**
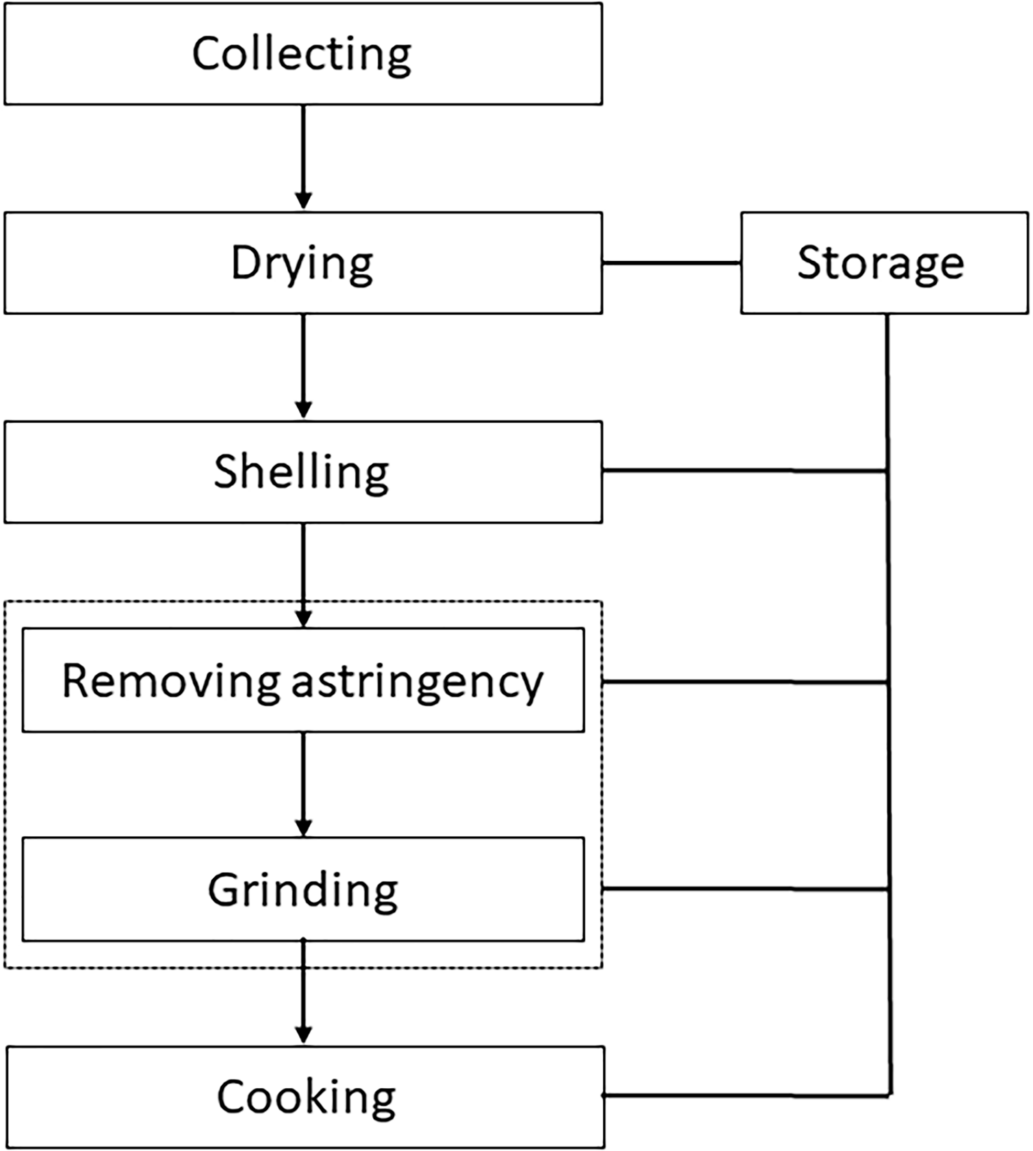
*Chaîne Opératoire* of acorn use in current papers. The procedures of removing astringency and grinding can be in reverse.

**Figure 4:**
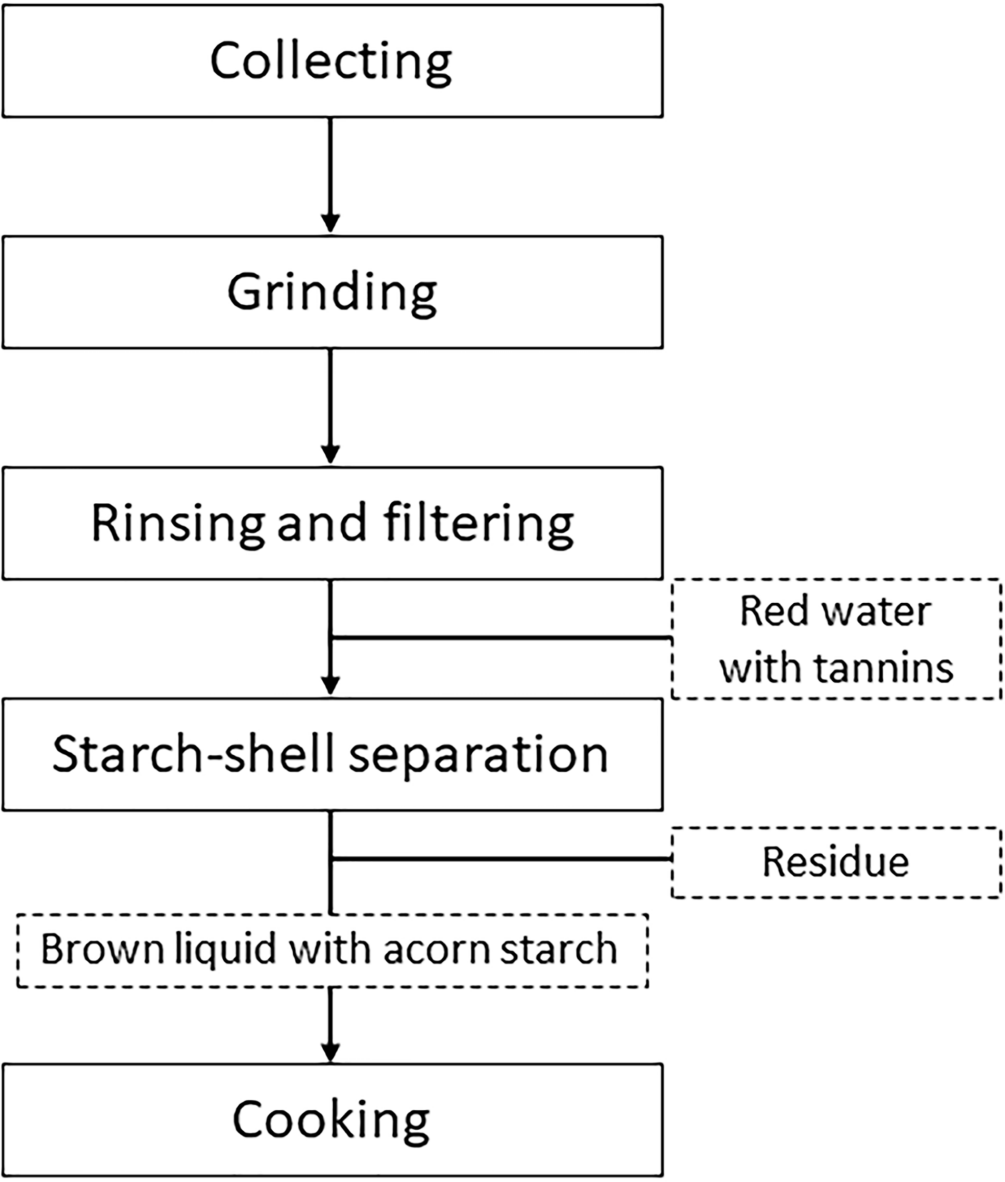
*Chaîne Opératoire* of ‘mook making’ in our study.

Acorn utilization as described in current papers (Qiao, 2010; Chen and Chen, 2019), often comprises collecting, drying, shelling, removing astringency, grinding and cooking (**Figure 3**). By contrast, the *Chaîne Opératoire* of ‘mook making’ in this case study is different (**Figure 4**). There was no shelling in our case. Also, astringency removal came after grinding, not beforehand. In the following, we discuss each sequence respectively.

Acorns ripen in the time when autumn turns into winter. Timely collection is essential so as to avoid acorns being damaged by insects or animals. There are two ways of collecting: first, to pick the ripe acorns without cupule on the ground; second, to pick the green acorns which have not fallen off. The taste of the two types of acorns is quite different (Chen and Chen, 2019). One can choose which method to take depending on harvesting conditions and culinary preference. In our case study, ripe acorns on the ground were chosen.

Acorns are often dried after collection (Muto, 2007; Chen and Chen, 2019), but not in our case study. According to previous literature, there are often three ways of drying: sun drying, frying and roasting (Jia et al., 1999), among which sun drying is the most common. The drying step is completed when the acorn shells crack.

In our case study, collected acorns are stored in a basket indoor for a short period before moving to the next procedure. In fact, acorn is very suitable for long-term storage. According to California ethnographies, acorns which are properly stored can last at least two years (Heizer and Elsasser, 1980; Pavlik et al., 1991). Acorns sometimes are mixed with soil and buried in pits (Primavera and Fiorentino, 2013). Alternatively, acorns are soaked in flowing rivers (Sip, 1986). There are three forms of acorn storage, namely whole acorn storage, storage of de-hulled acorns and storage of acorn flour (Sip, 1986). Meanwhile, acorns are often brined, soaked or boiled so as to extend the storage time (Sip, 1986).

The de-hulling method in this case was unusual, as the starch-shell separation was achieved by kneading after grinding. By contrast, the alternative way of de-hulling is to peel or crack the hard shells of acorns before grinding, right after harvesting and drying (Hosoya, 2011; Chen and Chen, 2019). The specific method and tools used for de-hulling depend on the number of acorns, acorn species (thickness of the acorn shell) and maturity (Barrett and Gifford, 1933; Spier, 1978). One way is using a small long stone to smash the acorns out of shells on a heavy flat stone slab (Qiao, 2010).

Both previous literature and our case study demonstrate that astringency removal is crucial for acorn utilization and is labor-intensive, while the actual procedures vary. After shelling, in some cases acorns are not ground. Whole acorns are soaked to remove astringency before being boiled or roasted for serving (Takahashi and Hosoya, 2002). In our case study, the fine starch was extracted by kneading after repeated leaching to remove tannins. Acorns can also be buried in mud with the purpose of removing tannins (Polimac et al., 2016). Sometimes there is no leaching or filtering after grinding (Bai et al., 2000).

As for cooking, despite regional variety, acorn flour or starch is often dissolved in water and the mixture is heated to make various foods, such as biscuits, tofu, noodles, jelly and soup (Zhang et al., 2014). Whole acorn can be boiled, steamed or pounded with other cereals (Tsuji, 1985; Nakui, 2006; Muto, 2007).

### Factors influencing nut-processing procedures

We have encountered some similarities and differences in acorn processing between our case study and that of existing studies. The processing procedure generally involves five stages: collecting, shelling, grinding, leaching and cooking (Matsuyama, 1982; Basgall, 1987; Tsuji, 1987; Tsuji, 1988; Takahashi, 1992; Hosoya, 2011; Primavera and Fiorentino, 2013; Chen and Chen, 2019; Wang and Jiang, 2022). Other nuts, such as chestnuts (*Castanea*) and horse chestnuts (*Aesculus*), require a similar procedure as acorns (Hosoya, 2011). However, the sequence of the procedures and the detailed methods differ, possibly due to different nut species and culinary tradition.

As for acid removal, different nut species require different methods. Some types of evergreen nuts, i.e. *Castanea*, are often consumed without removing astringency. Some other evergreen oaks, due to the low level of tannin content, their nuts only need simple procedures, such as water soaking (Matsuyama, 1982; Duanmu, 1995; Duanmu, 1997). As for acorns of deciduous oaks and horse chestnuts with high acid concentration, i.e. *Quercus acutissima* and *Aesculus chinensis*, they require comparatively sophisticated procedures to remove acid (Matsuyama, 1982; Nakui, 2006). For instance, horse chestnuts need to be soaked in hot water with ash for a day and night, so as to remove their high level of tannin content (Qiao, 2010). In our case study, the collected acorns belong to the species of *Quercus acutissima*, which contains high level of tannins. Laborious input is accordingly essential to remove tannins properly.

In addition to nut species, the choice of processing procedure is also affected by culinary requirement. Different acorn-processing procedures can occur in the same region with the very type of acorn. Depending on culinary requirements, typical nut processing methods can be divided into three types: starch extraction, whole-nut cooking and flour production (Tsuji, 1988; Takahashi, 1992). In our case study, it is about starch extraction. Pure starch and elaborate dishes can be produced but a relatively large amount of waste is inevitable (Takahashi and Hosoya, 2002). Notably, starch extraction as one particular form of acorn processing technique might have been East Asian specific and is rarely reported from other parts of the world. When it comes to whole nut cooking, for instance in Turkey, there is a custom that toasted and hulled acorns (*Quercus brantii*) can be eaten directly as a crunchy snack (Mason and Nesbitt, 2009). Whole-nut cooking is comparatively simple, while acid-removal is incomplete, resulting in a harsh taste. Otherwise, the method of flour production is the most common in many current papers (Primavera and Fiorentino, 2013; Chen and Chen, 2019. Whether toasted or not, hulled acorns can be ground into flour, which are then mixed with water to make bread, cakes and other foods (Primavera and Fiorentino, 2013; Polimac et al., 2016).

**Figure 5:**
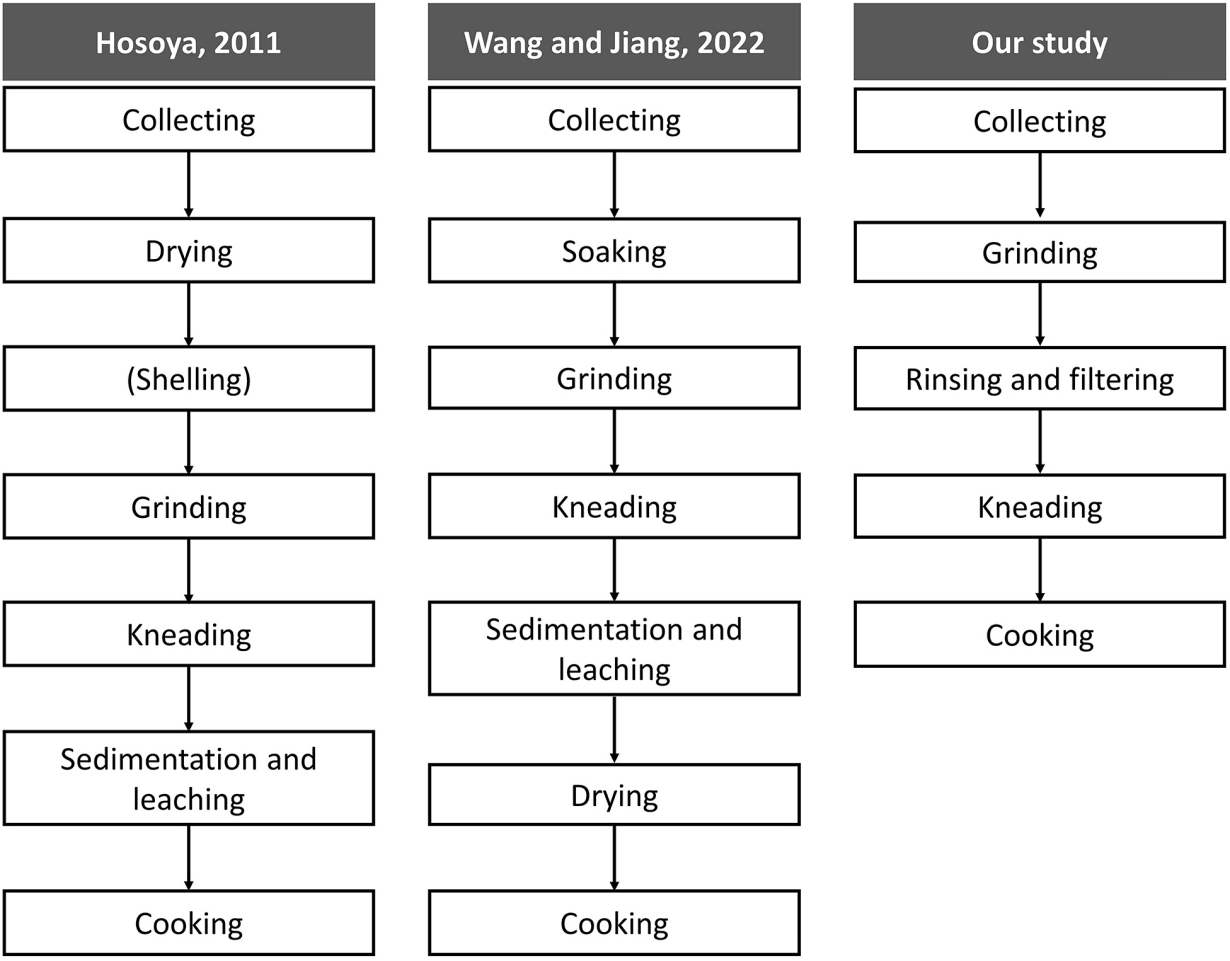
Comparison of three *Chaîne Opératoires* of starch extraction.

Nevertheless, sometimes different processing techniques can fulfill the same culinary purpose. For instance, starch extraction of acorns can be done in different ways. With the same purpose of extracting starch, Hosoya (2011) and Wang and Jiang (2022) demonstrate two alternative acorn processing procedures in East Asia (see **Figure 5**).

### Relating ethnographic observations to archaeological and archaeobotanical evidence

Our case study demonstrates one particular form of acorn processing in South Korea. Linking acorn-processing procedures with archaeological markers is conducive to the reconstruction of acorn use at prehistoric sites. Below we are to discuss potential archaeological and archaeobotanical markers associated with each step of the mook making process, starting from the collection up to the final product.

In our case, acorn collection takes place in a hilly area, close to settlement, where there are sawtooth oak trees. Existing literature suggests two plausible methods of tracking acorn collection which is off-site and sometimes can be difficult to be recognized. The first is site catchment analysis (Vita-Finzi et al., 1970; Qin et al., 2010), through which one may estimate whether acorn resources lie within the economic range of a settlement. Another is to detect the management of oak trees, for instance, the minimal use of oak amongst building timbers might have been associated with the preservation of oak trees (Fuller et al., 2011). By studying palaeo-ecological record such as pollen and charcoal influx, traces of fire can be detected, which is also argued to be related to oak management (Mason, 2000; Anderson, 2005; McCarthy, 1993).

In our case, jute sack is used for easy transportation. Carriers may also be other types of weaving products made of straw, bamboo, reed, etc. Collected acorns without cupules are stored in a loosely-woven basket, which presumably provides better ventilation, and kept in the kitchen. Such organic containers potentially are well preserved under waterlogged or mineralized conditions which are common at archaeological sites of Southeast China, South Korea and Japan. Other ethnographic studies demonstrate that acorn storage often takes place in granaries or pits (Kroeber, 1932; Mason, 1992). Indeed, acorn pits have been reported from prehistoric sites across East Asia (e.g. ZPICRA, 2004; Sun et al., 2007; Im and Lee, 2010; Kawashima, 2016). The location of storage pits varies from site to site, some under or near houses (Sun et al., 2007; ZPICRA, 2004) while others far away from settlements (Archaeological Institute of Kashihara, Nara Prefecture, 2000).

Grinding is often associated with specific tools. The modern crushing machines in our case are equivalent to tools such as slabs and handstones. By conducting starch and use-wear analysis, one is able to detect whether certain artefacts are ever used for processing acorns (Liu et al., 2010b; Hosoya, 2011). So far, acorn starch on grinding artefacts has been reported from more than 20 prehistoric sites in East Asia (e.g. Shibutani, 2009; Zhang, 2011; Liu et al., 2011; Ge et al., 2021).

Consistent with some existing literature (Barrett and Gifford, 1933; Nakui, 2006; Hosoya, 2011), our case study demonstrates that large amount of water is required to dissolve tannins. Rinsing and filtering is hence most likely to be completed away from household sites, but close to water, for instance by the river. As for containers of acorn powder while rinsing and filtering, in our case it is a modern jute sack, while according to Japanese ethnography, organic materials such as tree leaves are used, leaving few traces in archaeological context (Nakui, 2006).

Our case study also demonstrates that the practice of starch-shell separation is labor-intensive, and that such procedure results in finely fragmented residue. In other words, depending on the fragmented residue, one should be able to classify acorn remains and infer how it is originally processed (see **Table 2**). Different from whole-nut eating and flour production, it is possible to leave fragmented cotyledons in residue by starch extraction. Particularly, finely fragmented nutshells mixed with cotyledon pieces are likely to have been associated with the very method of starch extraction without shelling as shown in our case study, whereas large nutshells without cotyledons may indicate whole-nut eating or other processing techniques in which de-hulling precedes grinding. As for material culture associated with starch-shell separation, our observation indicates that containers such as basins are used for the process. In fact, such vessels are often reported from archaeological sites with acorn remains, for instance Shangshan site in Southeast China (ZPICRA, 2016; Jiang and Lei, 2016). Wang and Jiang (2022) argue that the Shangshan basins are used for acorn starch extraction, more specifically for starch leaching and drying, the procedure of which however is not witnessed in our case study. However, we argue that basins might have been used for starch-shell separation instead. As discussed earlier, the same culinary purpose, such as starch extraction, might have been fulfilled by different processing techniques (**Figure 5**). It is still to be addressed how precisely basins might have been involved in the acorn processing.

**Table 2:** Identification of different processing methods based on fragmented residue.

	Whole-nut eating	Flour production	Starch extraction (shelling)	Starch extraction (no shelling)
**Existence of fragmented cotyledons**	no	no	yes	yes
**Size of fragmented nutshells**	large	large	large	small

When it comes to cooking, because starch paste is easy to get burnt by the fire, food crust is likely to remain, leaving starch residue for identification. As a result, residue analysis of container vessels such as pots, jars and bowls can be crucial.

A summary of archaeobotanical and archaeological implication of each procedure of acorn mook making is presented in **Table 3**. Notably, the preservation chances of residues from each procedure of acorn processing vary in different conditions. Specifically, all products and by-products of acorn processing are likely to be well preserved under waterlogged or desiccated condition. In the case of carbonization, according to published reports (e.g., Schulz and Johnson, 1980; Pals, 1984; Mason, 1992), only fragments of acorn shells or cotyledons remains, whereas the products and by-products of acorn collection and storage which deals with whole acorn are ‘missing’ from archaeological sites. By comparison, even less traces of acorn processing can be preserved under mineralized condition.

**Table 3:** Archaeobotanical and archaeological evidences associated with each step of acorn mook processing.

Processing stage	Product	By-product	Micro-botanical evidence	Macro-botanical evidence	Archaeological evidence	Location where products/by-products are stored/discarded	Chances of preservation under carbonized condition	Chances of preservation under mineralized condition	Chances of preservation under waterlogged or desiccated conditions
**Collection**	whole acorns without cupules	cupules	acorn pollen and charcoal of oak trees	cupules	minimal use of oak amongst building timbers	woody area with oak trees	low	low	high
**Storage**	whole acorns without cupules		acorn starch	whole acorns	container vessels such as basket	pits under or near houses or away from settlements; in the flowing water	low	low	high
**Grinding**	acorn powder		use-wear and raw acorn starch on tools	fragmented acorn residue	grinding tools	dwelling sites	high	low	high
**Rinsing and filtering**	leached acorn powder		acorn starch	fragmented acorn residue	traces of constructed platforms near water for leaching	near water	high	low	high
**Starch-shell separation**	brown liquid containing raw acorn starch	fragmented shells and cotyledons	raw starch on tools	fragmented shells and cotyledons	basins	dwelling sites	high	low	high
**Boiling**	cooked acorn starch paste	charred acorn crust	gelatinized acorn starch in cooking vessels	charred acorn crust	heat-resistant pots and pyrotechnic structures	dwelling sites	high	low	high
**Solidification and serving**	cooked acorn starch paste		gelatinized acorn starch in serving utensils	acorn starch paste residue	serving utensils	dwelling sites	high	low	high

## Conclusion

Acorns are considered to have played an important role in human diet during prehistoric times. This paper tackles the *Chaîne Opératoire* of acorn utilization by conducting a case study of ‘mook making’ in South Korea based on an ethnological approach. Our case study of ‘mook making’ provides an explicit record of the *Chaîne Opératoire* of acorn utilization, which consists of collecting, grinding, rinsing and filtering, starch-shell separation, boiling, solidification and serving. In contrast with most current papers on acorn processing, our observation shows that the procedure of grinding sometimes precedes the procedure of de-hulling and acid removal. The difference is largely due to the culinary requirement, which in our case is for pure starch, rather than flour. Meanwhile, the processing procedure of acorns also depends on nut species. The higher content of tannins, the more complicated acid-removal process is in need. By discussing potential archaeological and archaeobotanical markers associated with each step of acorn mook making process, our case study brings new insights into archaeological interpretations of acorn remains. Particularly, we argue that finely fragmented nutshells mixed with cotyledon pieces are likely to have been associated with starch extraction, whereas large nutshells without cotyledons may indicate whole-nut eating or other acorn processing techniques in which de-hulling precedes grinding. Our study provides explicit reference for future study of acorn utilization in archaeological sites.

## Data availability statement

The raw data supporting the conclusions of this article will be made available by the authors, without undue reservation.

## Ethics statement

Ethical review and approval was not required for the study on human participants in accordance with the local legislation and institutional requirements. The patients/participants provided their oral informed consent to participate in this study.

## Author contributions

TA and MXT designed the study. JHA carried out field work. MXT and TA analyzed data, wrote and revised the manuscript. All authors contributed to the article and approved the submitted version.

## Funding

This work was supported by the National Social Science Fund of China (Grant No. 20CKG024), 2021-2022 Zhejiang University Global Partnership Fund, Fundamental Research Funds for the Central Universities and Zhejiang Provincial Scientific and Technological Innovation Project of College Students (Grant No. 2021R401176).

## Acknowledgments

We offer special thanks to Martin Jones for his invaluable suggestion to the revision of the paper. Thanks also to Okano Kirara for her help with Japanese documents. We thank Minyan He and Zhixuan Zhang for map making and translation. Aid in the case study was provided by our three participants from South Korea, whose help is gratefully acknowledged.

## Conflict of interest

The authors declare that the research was conducted in the absence of any commercial or financial relationships that could be construed as a potential conflict of interest.

## Publisher’s note

All claims expressed in this article are solely those of the authors and do not necessarily represent those of their affiliated organizations, or those of the publisher, the editors and the reviewers. Any product that may be evaluated in this article, or claim that may be made by its manufacturer, is not guaranteed or endorsed by the publisher.
